# Expression Profile of Defense Genes in Rice Lines Pyramided with Resistance Genes Against Bacterial Blight, Fungal Blast and Insect Gall Midge

**DOI:** 10.1186/s12284-018-0231-4

**Published:** 2018-07-13

**Authors:** Dhanasekar Divya, Kanaparthi Ratna Madhavi, Muralidharan Ayyappa Dass, Roshan Venkata Maku, Garladinne Mallikarjuna, Raman Meenakshi Sundaram, Gouri Sankar Laha, Ayyagari Phani Padmakumari, Hitendra Kumar Patel, Madamsetty Srinivas Prasad, Ramesh Venkata Sonti, Jagadish Sanmallappa Bentur

**Affiliations:** 1 Agri Biotech Foundation, Rajendranagar, Hyderabad, 500030 India; 2 ICAR-Indian Institute of Rice Research, Rajendranagar, Hyderabad, 500030 India; 3 CSIR- Centre for Cellular and Molecular Biology, Uppal Road, Hyderabad, 500007 India

**Keywords:** Resistance- gene pyramided lines- expression profiling- synergism- antagonism

## Abstract

**Background:**

Rice, a major food crop of the world, endures many major biotic stresses like bacterial blight (BB), fungal blast (BL) and the insect Asian rice gall midge (GM) that cause significant yield losses. Progress in tagging, mapping and cloning of several resistance (R) genes against aforesaid stresses has led to marker assisted multigene introgression into elite cultivars for multiple and durable resistance. However, no detailed study has been made on possible interactions among these genes when expressed simultaneously under combined stresses.

**Results:**

Our studies monitored expression profiles of 14 defense related genes in 11 rice breeding lines derived from an elite cultivar with different combination of R genes against BB, BL and GM under single and multiple challenge. Four of the genes found implicated earlier under combined GM and BB stress were confirmed to be induced (≥ 2 fold) in stem tissue following GM infestation; while one of these, cytochrome P450 family protein, was also induced in leaf in plants challenged by either BB or BL but not together. Three of the genes highlighted earlier in plants challenged by both BB and BL were also found induced in stem under GM challenge. *Pi54* the target R gene against BL was also found induced when challenged by GM. Though expression of some genes was noted to be inhibited under combined pest challenge, such effects did not result in compromise in resistance against any of the target pests.

**Conclusion:**

While R genes generally tended to respond to specific pest challenge, several of the downstream defense genes responded to multiple pest challenge either single, sequential or simultaneous, without any distinct antagonism in expression of resistance to the target pests in two of the pyramided lines RPNF05 and RPNF08.

**Electronic supplementary material:**

The online version of this article (10.1186/s12284-018-0231-4) contains supplementary material, which is available to authorized users.

## Background

Rice (*Oryza sativa* L.) is the major food for more than half of the world population. It is cultivated across the globe under diverse ecologies. Major biotic production constraints across these ecologies are bacterial blight (BB) caused by the bacterium *Xanthomonas oryzae* pv. *oryzae* (Xoo), blast (BL) caused by the fungus *Magnaporthe oryzae* (Mo) and the Asian rice gall midge (GM), *Orseolia oryzae*. While breeding for host plant resistance against the biotic stresses as the most desirable approach of their management is well recognized, recent progress in tagging, mapping and cloning of several of the resistance (R) genes against these pests has made this goal a lot more precise and easy. Specially the PCR based linked markers have enabled breeders to combine several R genes into a single cultivar through marker assisted backcross breeding without losing the features of the cultivar.

Against BB, 41 R genes have been reported so far: 29 dominant and 12 recessive; nine cloned and another nine mapped on to different chromosomes (see Zhang et al. 2017). In Indian context *Xa21*, *xa13* and *xa5* have been found effective (Sundaram et al. 2014). Closely linked or gene based markers are reported for these genes (Ronald et al. 1992; Sundaram et al. 2014; Hajira et al. 2016). Resistance against BL is reported to be conferred by over 100 genes including three recessive and 22 cloned genes (Sharma et al. 2016; Dong et al. 2017). Among these *Pi1*, *Pi2, Pi9* and *Pi54* are effective against a wide range of isolates of the pathogen in India (Krishnaveni et al. 2012). Linked markers for these genes have been reported (Tian et al. 2016; Madhav et al. 2005; Ramkumar et al. 2011). Against the Asian rice gall midge 11 genes have been reported including one recessive; of which eight have been mapped and three genes cloned (Bentur et al. 2016). Among these *Gm1, gm3*, *Gm4* and *Gm8* are effective against most of the seven prevailing biotypes in India (Bentur et al. 2011) and linked markers have been reported (Sundaram 2007; Dutta et al. 2014; Sama et al. 2014; Divya et al. 2015). There have been several successful attempts to introgress and pyramid these genes through marker assisted selection in elite genetic backgrounds. Sundaram et al. (2008) introgressed three BB resistance gene in the background of an elite variety Samba Mahsuri which formed the basic material for pyramiding other genes used in this study. These genes were later introgressed in different combinations into other elite cultivars like Triguna (Sundaram et al. 2009), parental lines of a hybrid Pusa RH10 (Basavaraj et al. 2010), Lalat (Das and Rao 2015), MTU1010 (Arunakumari et al. 2016), in both a maintainer line DRR17B, a restorer line RPHR-1005 (Balachiranjeevi et al. 2015; Kumar et al. 2017) and a set of three restorer and cytoplasmic male sterile lines (Ramalingam et al. 2017). Several reports are also available from other countries (Ruengphayak et al. 2015; Mi et al. 2018).

It is generally assumed that when such R genes are pyramided in a single plant, these act together to provide protection against all the target pests. However, some reports indicate antagonistic interactions among the R genes leading to compromise in resistance (Sundaram et al. 2009). To note such interactions among the R genes present studies were undertaken by us with 10 R gene pyramided rice lines in the genetic background of the popular elite cultivar Samba Mahsuri (BPT5204). In a parallel study we performed a microarray experiment to understand the cross talk between R genes under combined infection/infestation by BB, BL and GM (Maku et al. unpublished). This study identified a set of key genes which were observed to be induced under such combined challenge. In this paper, we have attempted to validate the expression of these genes at different time points and in different tissues under combined and individual challenges in order to gain better insights into the molecular crosstalk between the defense genes. Results revealed no distinct antagonism among gene expression leading to compromised resistance under combined threat.

## Results

### Resistance Against Target and Non-target Pests

The test lines were evaluated against the target pests BB, BL and GM under greenhouse conditions (Table 1). Nine of the test lines were resistant against BB. Against BL four of the lines were resistant while two were moderately resistant. Five lines were resistant to GM. Significantly, RPNF01, RPNF02 and RPNF03 were susceptible to BL despite presence of either *Pi2* or *Pi54* while RPNF06 was observed to be resistant with no *Pi* gene detected. Likewise, RPNF07 and RPNF09 were susceptible to GM despite presence of *Gm1* or Gm1 + *Gm4*.

**Table 1:** Rice lines with multiple R genes selected for the study and their reaction to the target pests under greenhouse

Line Code	Line designation	PCR reaction for the presence of R gene									Reaction against		
		BB			BL		GM				BB	BL	GM
		*Xa* *21*	*xa 13*	*xa* *5*	*Pi* *2*	*Pi* *54*	*Gm* *1*	*gm* *3*	*Gm* *4*	*Gm* *8*			
RPNF01	RP5922–21	+	–	+	–	+	+	–	–	–	R	S	R
RPNF02	RP5923–22	–	–	–	+	–	–	–	–	–	S	S	S
RPNF03	RP5924–23	+	–	+	–	+	+	+	+	+	R	S	R
RPNF04	RP5925–24	+	–	–	–	–	+	–	+	+	R	MR	R
RPNF05	RP5926–25	+	+	+	–	+	+	+	+	+	R	MR	R
RPNF06	RP5926–26	–	+	–	–	–	+	+	+	+	R	R	R
RPNF07	RP5871–1–8-6	+	+	–	+	–	+	–	–	–	R	R	S
RPNF08	RP5864–2–18-5	+	+	–	–	+	–	–	–	–	R	R	S
RPNF09	RP5872–5-156	+	+	–	+	+	+	–	+	–	R	R	S
RPNF10	Improved Samba Mahsuri (ISM)	+	+	+	–	–	–	–	–	–	R	S	S
RPNF11	Samba Mahsuri	–	–	–	–	–	–	–	–	–	S	S	S

*R* Resistant, *S* Susceptible, *MR* Moderately resistant

+ positive for presence of the functional allele

None of the test lines was resistant against non-target pests like sheath blight (ShB), rice tungro virus (RTV), brown planthopper (BPH), whitebacked planthopper (WBPH), rice leaffolder (LF) under greenhouse condition and against yellow stem borer (YSB) under natural field condition (Additional file 1: Table S1). Interestingly, two lines RPNF02 and RPNF05 recorded moderate resistance (damage score 3–6) against WBPH.

### Defense Gene Expression Under Combined Pest Challenge

#### Genes Involved in GM/BB Interaction

**Fig. 1:**
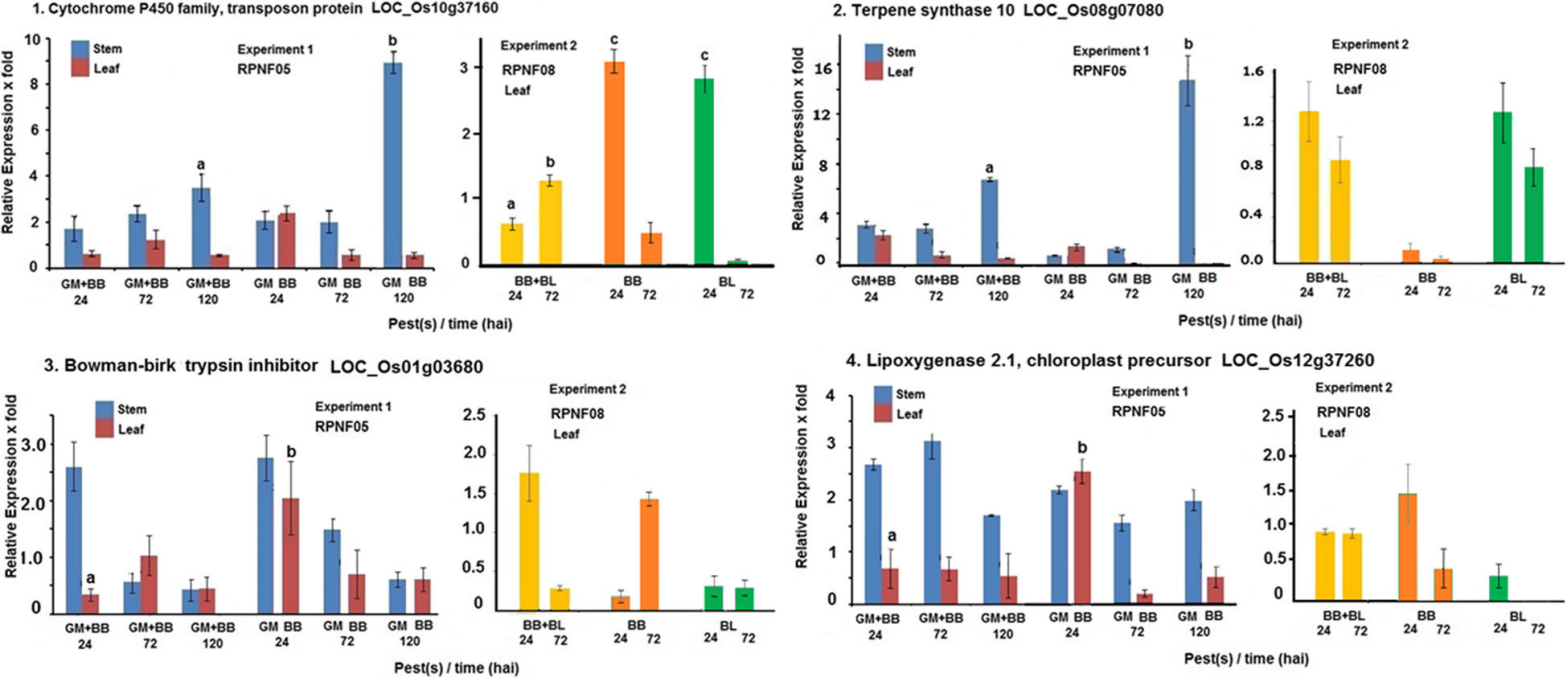
Relative levels of expression of the selected four defense related gene in rice line RPNF05 following challenge by BB and/or GM (Experiment 1) or in RPNF08 following BB and/or BL infection (Experiment 2). Columns (means ± SE) with different letter are significantly different (paired t test, *P* < 0.05)

All the four genes noted earlier to be associated with insect resistance viz. Cytochrome P450 family, transposon protein (LOC_Os10g37160); Terpene synthase 10 (LOC_Os08g07080); Bowman-birk trypsin inhibitor precursor (LOC_Os01g03680) and Lipoxygenase 2.1, chloroplast precursor (LOC_Os12g37260) showed significant induction (≥2 fold) in stem following GM infestation in Experiment-1 in RPNF05 (Fig. 1), though magnitude of induction was of lower order in comparison with the earlier microarray study (Table 2, Maku et al. unpublished). Terpene synthase expression was 15 fold high in GM infested plants at 120 h after infestation (hai) which was comparable to 18.7 fold noted in the earlier study. Expression of Bownman-birk trypsin inhibitor and of Lipoxygenase genes in leaf tissue was found induced above 2 fold at 24 hai by BB alone but not in plants subject to combined challenge of BB and GM. In contrast, in Experiment-2, these genes were not induced (< 2 fold) in leaf tissue of RPNF08 following BB and/or BL infection. Exception was Cytochrome P450 which was induced (≥2 fold) in leaf at 24 hai with BB or BL challenge but not together.

**Table 2:** Selected defense related genes used in validation studies and cDNA based primers

S. No.	FC^a^	Locus ID	Identity/Function	cDNA based primer sequences	Reference
1	22.71	LOC_Os10g37160	Cytochrome p450 family/induced upon defense response	F:GTTCTGCCTCCTCGTGAATA R:GGCTCGTGATGTAGATGAGC	^a^
2	18.72	LOC_Os08g07080	Terpene synthase 10, putative/secondary metabolism, volatile metabolites	F:GGCTCGAGTGAAGTACCAGA R:CAATGCAGAGAAGGTCGTTT	^a^
3	13.37	LOC_Os01g03680	Bowman-birk trypsin inhibitor/inhibits insect proteolytic enzymes	F:GACAAGGTGAAGTCGTGCTC R:TTAAGCTGGCTGGTTGTGAC	^a^
4	10.13	LOC_Os12g37260	Lipoxygenase 2.1, chloroplast/involved in JA biosynthesis	F:TGGAGCTGACGATAGAGGAC R:CACATAATCCGACACCCACT	^a^
5	4.87	LOC_Os01g65110	POT family protein, expressed/Induced in BL infected plants with *Pi54*	F:GTCGCCTTCTTCCTCTTCTC R:CAGATGCCATCATCATCAAC	^a,^ Gupta et al. 2011
6	4.71	LOC_Os07g03710	SCP-like extracellular protein/PR1, induced by Mo and Xoo infection (ref.)	F:GAAGTACGGCGAGAACATCT R:GTGGTCGTACCACTGCTTCT	^a^
7	3.05	LOC_Os01g71340	Glucan endo-1,3-beta-glucosidase/*PR2* Induced by fungal infection	F:GCAGACGTACAACCAGAACC R:GAACATGGCGAAAATGTAGG	^a,^ Balasubramanian et al. 2012
8	–	LOC_Os12g36830	*PR 10a*/involved in *Gm11* mediated resistance	F:ACCATCTACACCATGAAGCTTAAC R:GTATTCCTCTTCATCTTAGGCGTA	Rawat et al. 2013 Pruitt et al. 2015
9	–	LOC_Os10g01660	Isoflavone reductase	F:AGAAGAAGACGGGGAAGAAG R:GATCTCCGACTCCTGGATTT	Peng et al. 2015
10	–	LOC_Os11g42010 (AY914077)	*Pi54*/one of the pyramided genes	F:AAGATTTTCGAGGCTCTTCTCTA R:GATGAATCTGTTTCCTCGTCTTG	Rai et al. 2011
11	–	LOC_Os08g09670.1	*Gm4*/one of pyramided genes	F: CGCTTCAGACTGAGTCAACA R: CTTCCAATCCTTCATTGGTG	Divya et al. 2015
12	–	LOC_Os04g52970	*gm3* – one of the pyramided genes	F:TCTGGCCTGCACGAAGC R:GGCAAACGCCTACCCAGGA	Sama et al. 2014
13	–	LOC_Os08g15080	*Gm8 *– one of the pyramided genes	F:ATCGCCGCCAAGGCCGCGCT R:ATGATATGGGGGAGCAGCAT	Divya, 2016
14	–	LOC_Os11g45990	von Willebrand factor type A protein Involved in *Gm1* mediated resistance	F:AGTTTGTCATCAGGAAGCTTGCT R:GCTATATTCCTTGACGGGTCCAT	Rawat et al. 2012

^a^Designed for this study; − Not tested

#### Genes Involved in BL/BB Interactions

**Fig. 2:**
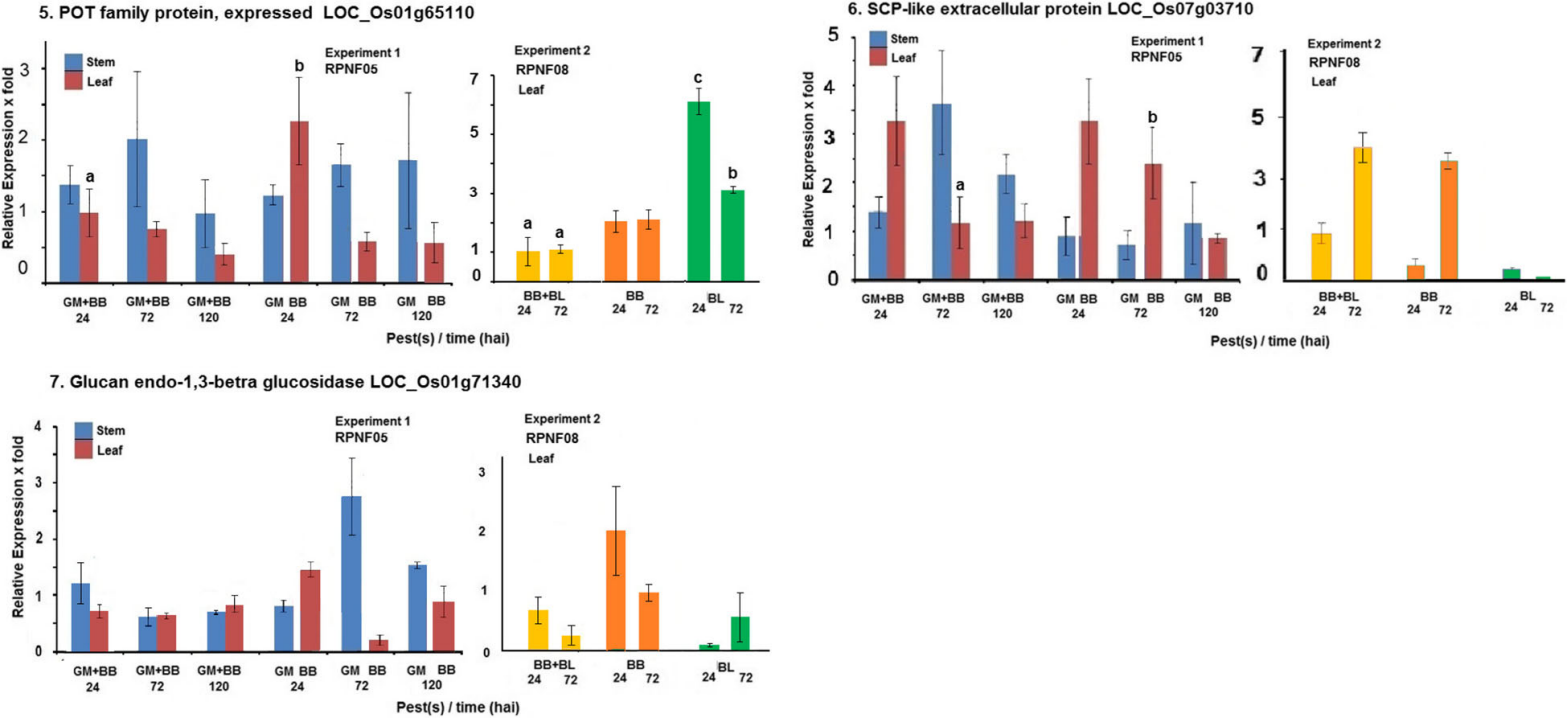
Relative levels of expression of the selected three defense related genes in rice line RPNF05 following challenge by BB and/or GM (Experiment 1) or in RPNF08 following BB and/or BL infection (Experiment 2). Column means ± SE with different letter are significantly different (paired t test, *P* < 0.05)

Three of the genes implicated in the earlier study to be associated with BB and BL infection in RPNF08 showed induction (≥2 fold) in leaf tissue in Experiment-2 (Fig. 2). POT family protein gene (LOC_Os01g65110) registered 6.1 and 3.1 fold increase in expression at 24 and 72 hai by BL; 2.0 and 2.1 fold at 24 and 72 hai by BB, respectively. These values were comparable with the earlier study (Table 2). Level of expression under combined infection by both the pathogens was lower than two fold at both time points. SCP like extracellular protein gene (PR1, LOC_Os07g03710) showed 3.9 and 4.3 fold induction in leaves at 72 hai by either BB alone or in combination with BL, while no induction was seen in leaves under BL infection alone. The third gene, Glucan endo-1,3-beta-glucosidase (LOC_Os01g71340) displayed 2.1 fold induction at 24 hai by BB, while BL infection or combined challenge did not induce the gene. Expression levels of these three genes in Experiment-1 in RPNF05 under GM and/or BB stress were highly variable among biological replications. Nonetheless, POT family protein gene showed 2 fold induction in leaves at 24 hai with BB; SCP-like protein gene showed 3.3 and 2.4 fold increase in leaf at 24 and 72 hai with BB alone and 3.3 fold at 24 hai with both GM and BB (Fig. 2). Interestingly, this gene and Glucan endo-1,3-beta-glucosidase were also found induced in stem tissue at 72 hai by GM.

#### Genes Involved in Rice BB Interaction

**Fig. 3:**
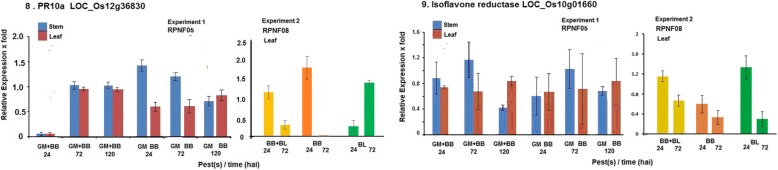
Relative levels of expression of *PR10a* and Isoflavone reductase in rice line RPNF05 following challenge by BB and/or GM (Experiment 1) or in RPNF08 following BB and/or BL infection (Experiment 2)

Two of the genes reported to be involved in rice-BB interaction, *PR10a* gene and Isoflavone reductase did not show induction (≥ 2.0 fold) in either of the experiments (Fig. 3).

#### Target Resistance Genes

**Fig. 4:**
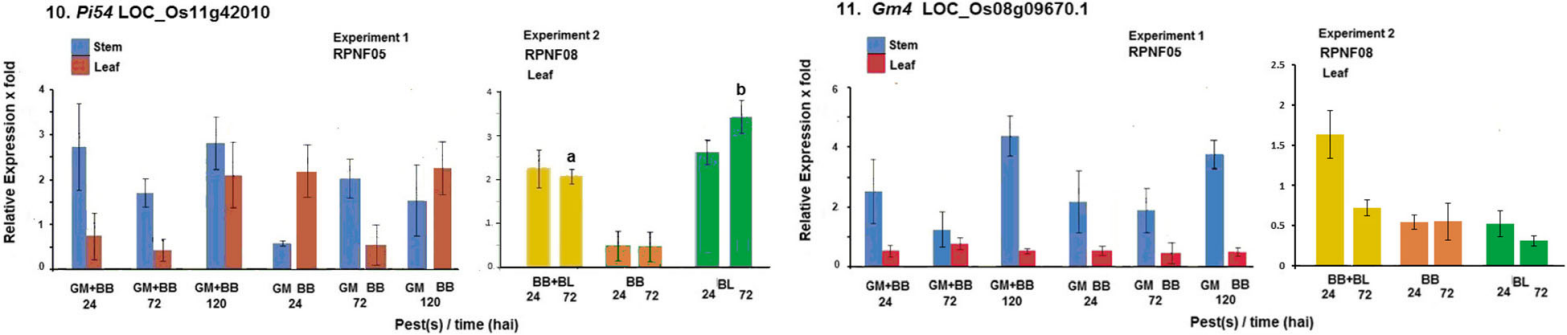
Relative levels of expression of *Pi54* and *Gm4* in rice line RPNF05 following challenge by BB and/or GM (Experiment 1) or in RPNF08 following BB and/or BL infection (Experiment 2). Column means ± SE with different letter are significantly different (paired t test, *P* < 0.05)

Expression of two of the target genes *Pi54* and *Gm4* was also noted in these two experiments (Fig. 4). *Gm4* expression level was significantly higher in stem, not in leaf, in RPNF05 at 24 and 120 hai by either GM alone or in combination with BB infection in Experiment-1 but it did not show any induction in Experiment-2. In contrast, expression of *Pi54* was found induced in both stem and leaf under combined or separate challenge by BB and GM in Experiment-1 while in Experiment-2 the gene was found significantly induced in leaf tissue at 24 and 72 hai either with combined infection of BB and BL or with BL alone, but not in plants challenged with BB alone.

**Fig. 5:**
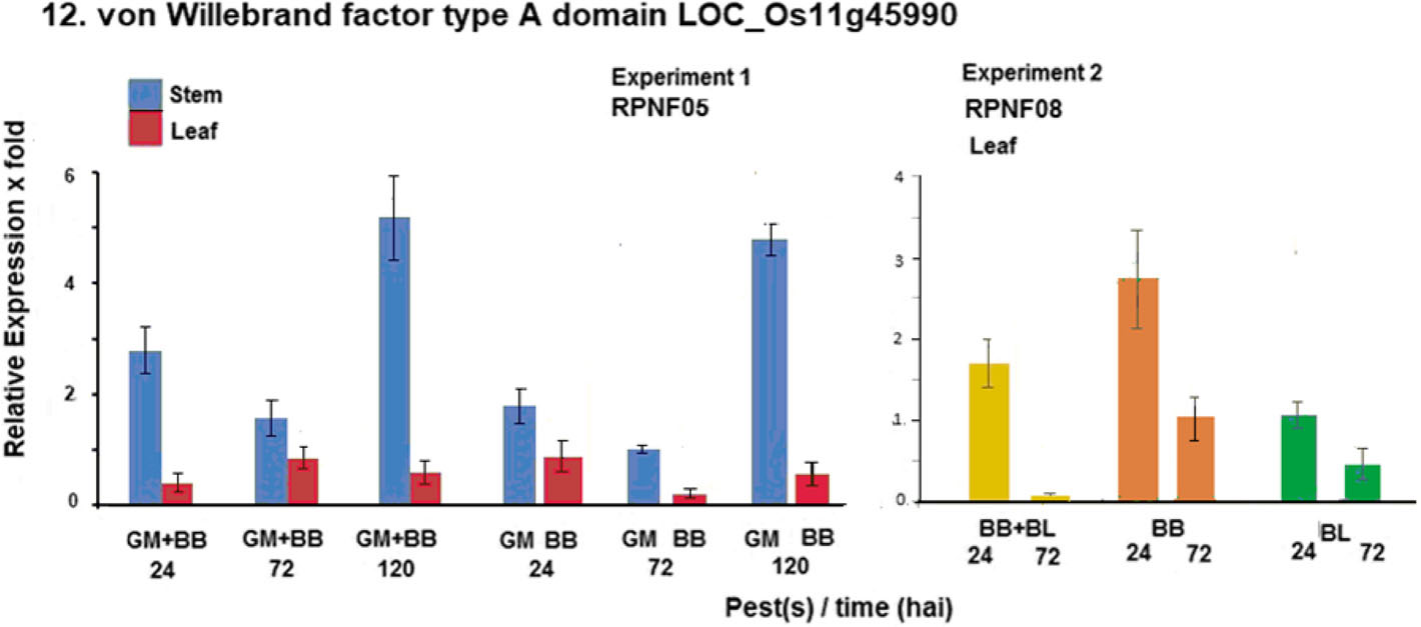
Relative levels of expression of von Willebrand factor type A domain protein gene in rice line RPNF05 following challenge by BB and/or GM (Experiment 1) or in RPNF08 following BB and/or BL infection (Experiment 2)

Another gene selected based on our earlier study, von Willebrand Factor type A, was also analyzed for its expression in both the experiments. Expression of the gene was highly induced in stem at both 24 and 120 hai with GM infestation with or without accompanying BB infection in Experiment-1 while it was also found induced in leaf at 24 hai by BB alone but not along with BL in Experiment-2 (Fig. 5). In addition, two more candidate genes *gm3* and *Gm8* were analyzed and these genes were not induced in both the experiments (Additional file 2: Table S2).

#### Sequential and Simultaneous Challenge on Expression of Resistance

An overview of the results highlighted significantly higher level of expression of Cytochrome P450 family protein and Terpene synthase in stem tissue at 120 hai in plants challenged with GM alone compared with those in plants challenged by BB and GM together (Fig. 1) suggesting negative effect of BB infection on the gene expression. On the other hand, expression levels of Lipoxygenase, POT family protein gene at 24 hai and of SCP like extracellular protein gene at 72 hai in leaf tissue of plants exposed to only BB were significantly higher than those in leaf tissue of the plants challenged simultaneously by both BB and GM (Figs. 1, 2), suggesting likely negative effect of gall midge infestation on expression of these genes. Significantly lower level of induction of *Pi54* in leaf tissue of plants at 72 hai- not at 24 hai– and POT family protein at both time points exposed to both BB and BL was observed as compared to those levels in plants infected with BL alone (Figs. 2, 4) suggesting probable negative influence of BB infection on expression of the genes. To investigate impact of such negative influence on resistance manifestation sequential infestation studies were conducted.

Exposing RPNF05 to GM, BB and BL in single, sequential or simultaneous exposure did not influence the resistance reaction against these pests (Table 3). Likewise, single, sequential or simultaneous infection of RPNF08 with BB and BL did not affect the resistance response of the line.

**Table 3:** Disease or pest reaction of the gene pyramided lines under sequential or simultaneous exposure to the pests

S. No.	Test line	Exposure on		Reaction to BB		Reaction to BL		Reaction to GM	
				Lesion length (cm)	Rating	Damage score	Rating	Plant damage (%)	Rating
		Day 1	Day 3	Mean ± SE		Mean ± SE			
1	RPNF05	BB	GM	1.51 ± 0.16	R			0	R
2		GM						0	R
3		GM + BB		1.83 ± 0.21	R			0	R
4		GM	BB	1.51 ± 0.16	R			0	R
5		BB		1.83 ± 0.22	R				
6		GM + BB + BL		1.53 ± 0.02	R	4.33 ± 0.33	MR	0	R
7	RPNF08	BL	BB	1.10 ± 0.04	R	1.6 ± 0.08	R		
8		BB		1.26 ± 0.01	R				
9		BL + BB		1.08 ± 0.04	R	1.76 ± 0.06	R		

*BB* Bacterial blight (*Xanthomonas oryzae *pv. *oryzae* – (IX020 strain), *BL* Blast (*Magnaporthae oryzae* – SP-28 strain), *GM* Gall midge (*Orseolia oryzae* – Biotype 1), *R* resistant, *S* Susceptible, *MR* Moderately resistant

## Discussion

Pyramiding of multiple R genes is often suggested as a strategy for durable and multiple pest resistance in crop plants. Tagging, mapping and cloning of several of the R genes conferring resistance against bacterial blight (BB), blast (BL) and gall midge (GM) in rice has led to identification of reliable molecular markers linked to the gene and gave a fillip to marker assisted selection and breeding for multiple pest resistance. Most often, *Xa21, xa13, xa5* conferring resistance to BB; *Pi1, Pi2, Pi9* and *Pi54* against blast and *Gm1*, *Gm4* and *gm3* against gall midge are involved in such breeding projects (Sundaram et al. 2008; Sundaram et al. 2009; Basavaraj et al. 2010; Das and Rao 2015; Arunakumari et al. 2016; Balachiranjeevi et al. 2015; Kumar et al. 2017; Ramalingam et al. 2017; Das et al. 2018). Specific gene based or closely linked PCR markers are available for these genes. Several popular elite rice lines have been ‘improved’ by incorporating some of these genes through Marker Assisted Backcross Breeding (MABB) involving both foreground and background selection (Kumar et al. 2017). Some of these studies have shown combined resistance to target pests like BB and BL or BB and GM (Kumar et al. 2017) in the gene pyramided lines under greenhouse controlled infection studies. It is assumed that pyramided genes act in unison and express combined resistance. Here we have examined this issue with greater clarity and with combination of multiple R genes against BB, BL and GM in ten gene pyramided lines developed in a common genetic background covering 14 defense related genes. We did not observe instances of distinct antagonism but recorded synergism among resistance pathways against these target pests.

Rice defense against BB, BL and GM has been fairly well studied in isolation. Interactions between rice and gall midge (Bentur et al. 2016; Sinha et al. 2017) display gene-for-gene interaction but with diversity mainly determined by the plant resistance gene and the insect biotype. Two of the R genes – *Gm1* and *Gm8*- confer HR independent (HR- type) resistance, rest of the known genes confer resistance through expression of HR (HR+ type) at the feeding site. In most of the cases, resistance is induced following pest attack, whereas *Gm1* mediated resistance appears to be novel and probably constitutive (Rawat et al. 2012). Global gene expression analysis through microarrays (Rawat et al. 2012; Agarrwal et al. 2016) or suppressive subtraction hybridization cDNA library (Rawat et al. 2013; Divya et al. 2016) revealed defense pathways similar to those reported for rice-pathogen interactions involving induction of cytochrome P450, phenyl propanoid pathway and pathogenesis related genes. However, exact copy of the gene involved was found varying widely.

Many recent studies have tried to elucidate early response of rice against blast fungus invasion through transcriptome analysis by microarrays or RNAseq (Jantasuriyarat et al. 2005; Bagnaresi et al. 2012; Gupta et al. 2011; Wei et al. 2013; Jain et al. 2017). A common pattern emerges from these studies that features the response into four phases like 1) initial oxidative burst involving ROS generating and quenching genes (NADP and other calmodulin dependent oxidases, peroxidases), 2) signal transduction involving receptor kinases, LRR motif protein genes that, 3) induce a host of transcription factors of WRKY family and others that modulate secondary metabolism and trigger salicylic acid (SA) and or jasmonic acid (JA) mediated signaling pathways leading finally to 4) expression of pathogenesis proteins that mark the induction of systemic acquired resistance. However, details of the specific genes and the time of their induction vary greatly depending on the genotype of the plant and pathogen being studied, involvement of specific R gene/genes and time lag. It is thus difficult to replicate the same pattern in every study. Mode of resistance conferred by the recessive gene *pi21* or the dominant *Pi33*, for instance, is altogether reported to be different (Vergne et al. 2007; Fukuoka et al. 2009; Vergne et al. 2010). Among the nine genes validated to be involved in *Pi54* mediated blast resistance in a transgenic Taipai309 rice line (Gupta et al. 2011) only one gene (endo-1,3-1,4-b-glucanase) was responsive in our study.

Accumulating evidence has revealed that the molecular mechanisms of rice resistance to BB are largely different from those of R protein-mediated resistance or effector-triggered immunity (ETI) (Zhang and Wang 2013) as noticed in the earlier two cases mentioned above. While 21 out of 22 cloned R genes against BL and two of the three cloned R genes against GM are NB-LRR class of R genes, only one out of nine cloned BB R genes encodes this type of protein. Rice genome contains 623–725 NB-LRR genes which are also implicated in resistance against other pests like BPH (Jing et al. 2017). The fact that eight of the nine cloned BB R genes encode different types of proteins suggests functional diversity in rice–BB interactions (Kuang et al. 2017). Resistance conferred by *Xa21* gene against BB in rice mediated by Receptor Kinase without HR is often dubbed as intense pattern triggered immunity (PTI) rather ETI. This gene primed several genes related to resistance and metabolism constitutively even prior to the pathogen attack (Peng et al. 2015). Resistance through recessive *xa13* and *xa5* is described as passive resistance since these two genes represent non-functional susceptibility genes; while *Xa13* allele is otherwise activated by the transcription activation like (TAL) effector proteins coded by Avr genes of the pathogen (Wang et al. 2014). Despite the above studies, there appears to be no information on interaction of such R genes when introgressed in a single plant. Present study attempted to address this lacuna in our knowledge.

In our concurrent study (Maku et al. Unpublished) we subjected the transcriptome from the two experiments to microarray analysis to identify sets of genes that are either upregulated or down regulated in the two gene pyramid rice lines RPNF05 (Experiment-1) and RPNF08 (Experiment-2). These lines were subjected to simultaneous challenge by BB and GM or BB and BL, respectively. Of the ten genes detected in Experiment-1 with more than 10 fold upregulation, four genes were selected based on basis of the earlier report of their involvement in plant defense. Likewise, of the 16 genes detected in Experiment-2 with more than three fold upregulation three genes were selected in the present study. In addition, seven more relevant genes were also included in the study. Gene expression validation in the present study was more elaborate, under similar format for the Experiment-1 and Experiment-2, involving separate evaluation for single or combined infection, for each of the tissues sampled and for each of the time points of tissue sample collection. As mentioned above, results of the present studies, in general, corroborated the earlier study.

Interestingly, when the ten pyramided lines were evaluated against the three target pests, their response was not always in agreement with the PCR detection of the target genes. While BB reaction agreed with presence of one of the *Xa* genes, two of the test lines (RPNF07 and RPNF09) were found susceptible to GM despite the PCR detection of *Gm1* or *Gm4* gene. Likewise, RPNF01, RPNF02, RPNF03 were observed to be susceptible to BL though these lines had shown presence of *Pi2* or *Pi54,* respectively. We attribute these results to possible false positive results of PCR test based on poorly linked *Gm1* markers (Biradar et al. 2004) or lack of specific gene donors for *Pi2* (C101A51) or *Pi54* (Tetep) in the pedigree of RPNF01, RPNF02 or RPNF03, respectively. In contrast, RPNF04 and RPNF06 displayed BL resistance despite apparent lack of Pi gene. We suspect possible role of one of the Gm genes present in these lines to have provided cross resistance to BL. However, such speculation needs more studies for confirmation.

Among the four genes implicated in plant-insect interactions, Cytochrome P450 family, transposon protein (LOC_Os10g37160) is a member of the large family of genes. Plant genes of this family are also reported to be involved in herbicide tolerance (Xu et al. 2015), chemical defense and hormone biosynthesis. In our earlier study another copy of Cytochrome P450 gene (Os03g0658800) was found upregulated five fold in rice variety Suraksha with *Gm11* gene at both 24 and 120 hai with GM biotype1 (Rawat et al. 2013). In contrast, another Cytochrome P450 protein coding gene (LOC_Os03g45619) was noted to be upregulated in rice variety Kavya with *Gm1* gene at 24 hai with compatible GM biotype 4 but not with incompatible GM biotype1 (Rawat et al. 2012). In agreement with this, another gene (LOC_Os03g04530) was also reported to be down regulated in rice line RP2068–18–3-5 with *gm3* gene infested with GM during compatible interaction as compared with uninfested plants (Agarrwal et al. 2016). Present results also underscored the role of Cyp450 genes in rice-GM interaction. Moreover this gene was also found upregulated in leaf tissue of the test line RPNF08 at 24 hai with BB or BL but not when subjected to combined infection. One of the CYP450 genes (LOC_Os06g39780) was induced 16 fold in rice line PB1 with *Pi9* gene at 24 hai with BL infection (Jain et al. 2017). Thus, it may be noted that many different copies of the CYP gene family are involved in plant defense against pests while their expression pattern is reported to differ based on plant and pest genotypes under the study.

Terpene synthase genes are involved in secondary metabolism and synthesis of volatile metabolites or phytoalexins as defense response to insect pests or pathogens (Bohlmann et al. 1998). Two rice STPS genes viz. LOC_Os04g27430 and LOC_Os08g07100 are reported to be induced by BPH feeding and influence antixenosis in rice Rathu Heenati (Kamolsukyunyong et al. 2013). The specific gene under study, LOC_Os08g07080 was found induced following fall armyworm feeding on leaves of Nipponbare japonica rice (Yuan et al. 2008) and also reported to be involved in detoxification of auxin-type herbicide quinchlorac (Xu et al. 2015).

The third gene, Bowman-birk trypsin inhibitor precursor, putative, is likely to code for trypsin inhibitors targeting digestive trypsin of the feeding maggot. Many protease inhibitors of plant origin have been reported with insecticidal activity and genes coding for these have been extensively used in plant transformation to provide insect and pathogen resistance (Ryan 1990). Overexpression of RBBI2–3 in transgenic rice plants resulted in resistance to the BL pathogen (Qu et al. 2003).

Lipoxygenases are involved in biosynthesis of jasmonic acid (JA) and other volatiles. A gene encoding chloroplast-localized 9-LOX, Osr9-LOX1, from rice, was induced by SA and increased stem borer resistance (Zhou et al. 2014). As one of the JA biosynthesis genes LOC_Os12g37260 (OsLOX2; 2) was observed upregulated in cold tolerant rice line as compared with cold sensitive line under cold stress (Yang et al. 2015) and also in drought tolerant introgressed line under drought stress (Huang et al. 2014). On the other hand, during rice (Kavya)-gall midge interaction another Lipoxygenase gene (LOC_Os08g39850) was not found induced or suppressed despite being picked up through microarray analysis (Rawat et al. 2012). Two of the lipoxygenases picked up from microarray analysis, lipoxygenase 2 (LOC_Os03g52860) and lipoxygenase 2.1 (LOC_Os12g37260) did respond to GM infestation in rice RP2068–18–3-5 (Agarrwal et al. 2016).

One of the genes tested, *PR10a*, was earlier reported to be a key gene in conferring gall midge resistance in rice variety Suraksha having *Gm11* gene (Rawat et al. 2010). However, in the present study this gene was not induced in rice line RPNF05. The target gene *Gm4* in this line displayed 2 to 4 fold upward induction in stem tissue at 24 hai as earlier characterized (Divya et al. 2015). This induction was not affected by simultaneous infection of the plant with BB pathogen. Two other genes, *Pi54* (LOC_Os11g42010) and von Willebrand factor type A protein (LOC_Os11g45990) were also found induced in stem tissue following GM infestation. While the latter has been reported to be induced during both compatible and incompatible interactions between Kavya rice and GM (Rawat et al. 2012), former gene was never examined for its role in this context. Significantly, analysis of another SSH cDNA library developed from Aganni rice with *Gm8* gene, highlighted a list of 27 genes that were distinctly upregulated after GM infestation as compared to uninfested plants but the list did not contain any of the above seven genes (Divya et al. 2016).

Among the three genes selected based on their induction in Experiment-2 in microarray study (Maku et al. unpublished), Glucan endo-1,3-beta-glucosidase (LOC_Os01g71340), a member of PR2 class of genes, was found induced in leaves with BB infection alone. This gene was reported to be induced in a NIL of rice line PB1 with *Pi9* gene following infection with blast pathogen (Jain et al. 2017) and in other rice BL interactions (Balasubramanian et al. 2012). Microarray analysis of transcriptomes of one blast susceptible and two blast resistant lines with *Pi1* or *Pi9* gene revealed that the genes involved in signaling, secondary metabolism and those encoding WRKY transcription factors, were among up-regulated genes (Wei et al. 2013). Overexpression of one of the WRKY gene, OsWRKY47, greatly enhanced rice resistance to blast. None of the early induced transcription factor genes were picked up in our microarray study (Maku et al. unpublished), possibly because of pooling of tissue samples across time points, and hence not included in this validation study.

The POT (proton-coupled oligopeptide transporter) family gene is reported to be involved in rice response to potassium deficiency (Shankar et al. 2013) and over expressed under Fe-deficient conditions (Zheng et al. 2009). It is reported to be induced in plants with *Pi54* gene following BL infection (Gupta et al. 2011).

Few studies have examined transcriptomic responses to different biotic stresses in a parallel way. In a study Narsai et al. (2013) observed response to viral infection distinctly different from the response to bacterial, parasite and fungal infection, with fewer functional categories showing overlapping responses.

## Conclusions

Marker assisted backcross breeding has enabled pyramiding of multiple resistance genes into any single elite genetic background aimed at multiple and durable pest resistance. However, no detailed information is available on possible molecular cross talks among defense pathways in rice in such gene pyramids. Our studies covering 11 related advanced breeding lines with a uniform elite genetic background of BPT5204 involving introgression in different combination of nine R genes and subjecting them to single, sequential and simultaneous challenge of target pests BB, BL and GM revealed induction of several defense genes in response to more than one pest attack. Cross response of *Pi54* and *Gm4* was also suggested and inhibition of expression of *Pi54* by other target genes was indicated. Nonetheless, no distinct antagonism was seen in two of the test lines RPNF05 and RPNF08 in conferring resistance to BB, BL and GM. More studies are needed to resolve possible antagonism in other test lines.

## Methods

### Rice Lines Used

Eleven rice lines designated as RPNF01 through RPNF11 were used in this study. These lines were derived from a common elite variety BPT5204 (Samba Mahsuri- RPNF11- susceptible to BB, BL and GM) through systematic introgression of any of the three BB resistance genes *Xa21, xa13, xa5;* two of the BL resistance genes *Pi2*, *Pi54* and four of the GM resistance genes*, Gm1, gm3, Gm4* and *Gm8* in different combinations through marker-assisted backcross breeding (MABB) (Sama et al. 2014; Madhavi et al. 2016; Kumar et al. 2017) as detailed in Table 1. Foreground selection for introgression of these genes was accomplished using reported linked or gene based markers (Additional file 3: Table S3). In the microarray study, two of these lines viz., RPNF05 and RPNF08 were subjected to combined infection of BB + GM (hereafter as Experiment-1) or BB + BL (hereafter as Experiment-2), respectively, and target tissues i.e. basal part of stem (for GM) and terminal part of leaf (BB and BL) were collected at different time points after challenge and pooled to analyze expression profiles of key response genes (Maku et al. unpublished).

### Defense Genes Identified

These studies identified more than 10 genes with > 10 fold upregulation and another ten genes with > 10 fold downregulation in Experiment-1, while 15 gene displayed > 3 < 10 fold upregulation in Experiment-2. Of these, a set of seven genes; four from Experiment-1 and three from Experiment-2 was selected based on significant level of induction following combined challenge and earlier reports of the gene being involved in plant defense against biotic stresses. In addition, two genes reported to be involved in rice-BB interaction and five target genes in the pyramided lines were also selected (Table 2) in the present study.

### Phenotyping

Test lines were evaluated against target pests like BB, BL and GM and against non-target pests like sheath blight (ShB), rice tungro virus (RTV), brown planthopper (BPH), white backed planthopper (WBPH), leaffolder (LF) and yellow stem borer (YSB) under greenhouse and/or field condition as per the standard evaluation system for rice (International Rice Research Institute (IRRI), 2013). These methods are briefly described hereunder.

Against BB the selected lines including the susceptible (i.e. Samba Mahsuri: RPNF11) and the resistant (i.e. Improved Samba Mahsuri: RPNF10) checks were screened using a local virulent isolate of *Xoo*, viz., IX020 through clip inoculation method (Kauffman et al. 1973). The isolate belongs to pathotype 4 and is avirulent against *xa13* gene while being moderately virulent against *Xa21* (Yugander et al. 2017). The bacterial pathogen was multiplied on modified Wakimoto’s Agar (MWA) and a bacterial suspension (ca. 10^9^cfu/ml) was prepared using 3-day old culture. Test and control plants were raised in plastic trays (60 X 40 X 7 cm) in glasshouse and when the plants were 21 days old these were inoculated. Inoculation was done by cutting individual leaves 5 cm from tip using a sterile scissor dipped in a freshly prepared bacterial suspension. Inoculated plants were scored as per standard evaluation system (SES) scale for rice (International Rice Research Institute (IRRI), 2013). In case of simultaneous BB and GM challenge test plants were pre-exposed (4 days prior) to gall midge adults for laying of eggs and their incubation. Eggs would hatch on 5th day which was also the time for BB infection.

Uniform blast nursery protocols were followed for evaluation of test lines against *Mo*. The seeds of the test lines were sown on uniform blast nursery (UBN) bed along with resistant (Tetep) and susceptible (HR12 and BPT5204-RPNF11) check lines. The lines were sown in a single row of 50 cm long and 10 cm between the rows. The susceptible check HR12 was sown as border row all around to spread the inoculum. After 15 days of germination, the plants were inoculated with most virulent isolate (SP-28) maintained at IIRR (Prasad et al. 2009). The spore suspension (10^5^ conidia/ml) was sprayed with help of a glass atomizer. To facilitate heavy infection, high humidity was maintained by an automatic mist maker and then covering the nursery beds with polythene sheets. Inoculated seedlings were monitored for the development of blast lesions 15 days after inoculation. The plants were scored and evaluated as per standard evaluation system (SES) scale for rice (International Rice Research Institute (IRRI), 2013).

For Experiment-2 RPNF08 plants were raised in plastic cups (500 mL) along with plants of RPNF10 and RPNF11 and resistant check Tetep and susceptible check HR12. After 15 days at four-leaf-stage, rice blast pathogen (*Mo*, Strain no: SP-28) spore suspension was sprayed to inoculate the plants. Control set of plants were mock inoculated by spraying water only. After inoculation, the plants were transferred to high humidity chamber. One set of plants was simultaneously clip inoculated with BB suspension for simultaneous challenge. The leaf samples were collected at 24 and 72 hai with blast and/or BB and from mock inoculated control plants. The samples thus collected were stored in liquid nitrogen for further studies. Symptoms begin to appear 72 hai and the disease intensity was scored after 15 days of inoculation in a spare set of inoculated plants.

Greenhouse screening of test lines against GM was done using gall midge biotype 1 (GMB1). Test plants along with suitable controls were raised in trays and 21 day old plants were exposed to freshly emerged flies (30 females and 10 males) obtained from nucleus culture under mesh cage for 48 h for oviposition. Infested plants in the trays were later transferred to high humidity chamber and left for two days for the eggs to hatch. On 5th day after release of adults, plants were examined for the presence of maggots at apical meristem which was considered as ‘0 day’ of larval infestation. For Experiment-1, as described above, one set of plants (RPNF05) were used for BB inoculation on day 5 for simultaneous challenge. Basal part of the stem up to 2 cm above the soil, along with leaf tissue when specified, was cut from the infested plants and stored immediately in liquid nitrogen for RNA isolation at a later stage. Some of the treated plants were left for observation of plant damage. Plants were scored for gall midge damage when more than 90% of the TN1 plants (susceptible) showed gall development. Performance of the entry was rated on basis of percentage plant damage. Plants with 0–10% plant damage are graded as resistant (R) and greater than 10% as susceptible (S). In case of simultaneous challenge by BB, BL and GM (Table 3, S.No. 6), test plants were exposed to GM adults four days prior for oviposition; on 5th day (day newly hatched maggots reach feeding site) these plants were infected with BL spores and BB by leaf clipping method as described above.

#### Gene Expression Under qRT-PCR

The selected defense gene expression under single, simultaneous or sequential challenge by BB, BL or GM was noted in two experiments identical to the concurrent study (Maku et al. unpublished).

In Experiment-1 RPNF05 test plants were raised in standard 3 L pots and when these were 25 days old, one of the three subsets of pots was exposed to gall midge (GM), another was subject to leaf clipping to inoculate with BB (strain IX020) and the third set was simultaneously challenged with both GM and BB. Three replications were maintained for each of the above three treatments and one set of uninfested/mock inoculated control set was also maintained. Tissue samples were collected from 20 to 30 leaves and basal part of stem after 24, 72 and 120 hai and used for total RNA isolation.

In Experiment-2, RPNF08 test plants were used. Test plants were raised in 500 mL plastic cups and when these were 25 days old, one of the three subsets of pots was subjected to blast (SP-28 strain), another was subject to leaf clipping to inoculate with bacterial blight (BB-strain IX020) and the third set was simultaneously challenged with both blast and BB. Three replications were maintained for each of the above three treatments while one set of uninfested / mock inoculated control pots was also maintained. Leaf tissue samples were collected from 20 to 30 leaves after 24 and 72 hai for total RNA isolation.

About 3 μg of RNA was used for first-strand cDNA synthesis using the iScript cDNA synthesis kit (Bio-Rad, USA) following the manufacturer’s guidelines. Real Time PCR was performed using CFX96 Real Time PCR System with the SYBR green chemistry (Bio-Rad, USA) according to the manufacturer’s instructions. Rice ubiquitin gene, OsUbq (GenBank accession no. AK059694), was used as the endogenous control. Real Time PCR reaction volume of 10 μl contained 5 μl SYBR Green PCR Master Mix (Bio-Rad, USA), 500 nM each of forward and reverse primers and 30 ng of the cDNA samples. To calculate mean relative expression levels, cDNAs from three independent biological samples in two technical replications each were used. PCR was initiated with denaturation at 95 °C for 5 min followed by 40 cycles of denaturation at 95 °C for 10s and annealing and extension at 60 °C for 30s. A melt curve analysis was done to determine the specificity of the reaction. After normalization, quantity of each mRNA was calculated from the threshold points located in the log-linear range. The data from different PCR runs or cDNA samples were compared by using the mean of CT values of the three biological replicates that was normalized to the mean of CT values of the endogenous gene. The relative standard curve method was used for the quantification of mRNA levels and displayed as Relative Expression Values (REV). Expression ratios were calculated using the 2^-∆∆Ct^ method (Livak and Schmittgen 2001). The data were analyzed using the Bio-Rad CFX Manager 3.1 Software (Bio-Rad, USA) with default baseline and threshold. Relative transcription levels are presented graphically. All the 14 identified genes were validated in leaf and stem tissues of the plants separately for the two experiments at each of the time points. Results are presented as mean ± SE of relative expression in comparison with corresponding uninfested control sample. Means higher than 2 fold value of relevant treatments were compared through paired ‘t’ test at *P* < 0.05.

## Additional files

Additional file 1: **Table S1.** Phenotypic evaluation of the 11 test lines against non-target pests in greenhouse and field tests. (XLSX 12 kb)

Additional file 2: **Table S2.** Quantitative Reverse Transcription PCR data against 14 genes recorded in Experiment-1 and Experiment-2. (XLSX 28 kb)

Additional file 3: **Table S3.** Details of primer pairs used in detecting pyramided target genes in test lines. (XLSX 10 kb)

## Abbreviations

BB: Bacterial blight
BL: Blast
BPH: Brown planthopper
ETI: Effector triggered immunity
GM: Gall midge
HR: Hypersensitive reaction
JA: Jasmonic acid
LF: Leaf folder
MABB: Marker assisted backcross breeding
*Mo*: *Magnaporthe oryzae*
NB-LRR: Nucleotide binding-leucine rich repeat
POT: Proton-coupled oligopeptide transporter
PR: Pathogenesis related protein
PTI: Pattern triggered immunity
R: Resistance
REV: Relative expression values
ROS: Reactive oxygen species
RTV: Rice tungro virus
S: Susceptible
SA: Salicylic acid
SES: Standard evaluation system
ShB: Sheath blight
TAL: Transcription activation like
UNB: Uniform blast nursery
WBPH: Whitebacked planthopper
*Xoo*: *Xanthomonas oryzae pv. oryzae*
YSB: Yellow stem borer
